# Patient and public involvement in implementation of evidence-based guidance for musculoskeletal conditions: a scoping review of current advances and gaps

**DOI:** 10.1186/s41927-022-00310-x

**Published:** 2022-10-22

**Authors:** Opeyemi O. Babatunde, Shoba Dawson, June Brammar, Linda Parton, Krysia Dziedzic, Adewale O. Adebajo

**Affiliations:** 1 School of Medicine, Keele University, Keele, Staffordshire ST5 5BG UK; 2 Centre for Academic Primary Care, Population Health Sciences, Bristol Medical School, University of Bristol, Bristol, UK; 3 Haywood Academic Rheumatology Centre, Midlands Partnership NHS Foundation Trust, Stoke on Trent, ST6 7AG UK; 4 Faculty of Medicine, Dentistry and Health, University of Sheffield, Sheffield, UK

**Keywords:** Musculoskeletal conditions, Guidelines, Evidence-based recommendations, Patient and public, Involvement, Engagement, Implementation, Knowledge mobilisation

## Abstract

Advances in musculoskeletal (MSK) research have been successfully curated into widely endorsed evidence-based recommendations and guidelines. However, there continues to exist significant variations in care and quality of care, and the global health and socio-economic burdens associated with MSK conditions continues to increase. Limited accessibility, and applicability of guideline recommendations have been suggested as contributory factors to less than adequate guideline implementation. Since patient and public involvement (PPI) is being credited with increasing relevance, dissemination and uptake of MSK research, the success of guidelines implementation strategies may also be maximised through increasing opportunities for PPI input. We therefore conducted a scoping review of literature to explore PPI in implementation of evidence-based guidance for MSK conditions. A comprehensive search was used to identify relevant literature in three databases (Medline, Embase, Cinahl) and two large repositories (WHO, G-IN), supplemented by grey literature search. Eligibility was determined with criteria established a priori and narrative synthesis was used to summarise PPI activities, contexts, and impact on implementation of MSK related evidence-based guidance across ten eligible studies (one from a low-and middle-income country LMIC). A prevalence of low-level PPI (mainly consultative activities) was found in the current literature and may partly account for current experiences of significant variations and quality of care for MSK patients. The success of PPI in MSK research may be lessened by the oversight of PPI in implementation. This has implications for both high- and low-resource healthcare systems, especially in LMICs where evidence is limited. Patient and public partnership for mobilising knowledge, maximising guideline uptake, and bridging the research-practice gap particularly in low resource settings remain important and should extend beyond PPI in research and guideline dissemination activities only. This review is a clarion call to stakeholders, and all involved, to transform PPI in MSK research into real world benefits through implementation approaches underpinned by patient and public partnerships. We anticipate that this will enhance and drive quality improvements in MSK care with patients and for patients across health and care settings.

**Supplementary Information:**

The online version contains supplementary material available at 10.1186/s41927-022-00310-x.

## Introduction

Over the past two decades, musculoskeletal (MSK) conditions including back pain and arthritis have remained the leading cause of disability worldwide [1–3]. Coupled with an ageing population and multimorbidity clusters, the burden of musculoskeletal pain is increasing in high- and also in Low- and Middle-income countries (LMICs) [1–6]. Despite recent advances in rheumatology and MSK research, management of most patients with MSK conditions is yet to be at par with current best evidence especially in low resource settings [5, 6]. The substantial health [1–4, 6] and socioeconomic costs [1, 3, 4] attributable to MSK conditions contribute to the growing need to improve care quality and minimise significant variations in care using current best evidence [1, 5].

Evidence-based recommendations provide clinical guidance and advice and have the potential to improve health and social care for people with MSK conditions. Such guidance, usually produced by internationally recognised organisations (e.g., National Institute for Health and Care Excellence (NICE), Osteoarthritis Research Society International (OARSI), the European League Against Rheumatism (EULAR), and American College of Rheumatology (ACR), is often underpinned by collaborative efforts of researchers, healthcare professionals, and patients and public involvement (PPI). However, there is evidence to suggest low uptake, application [7], and poor adherence [8] to these guidelines, and that recommendations do not always influence patient care and practice in real world settings [8].

Numerous strategies [7, 8] including clinician educational meetings, barrier analysis studies, dissemination of printed guidelines and patient brochures have been proposed and are being used to introduce guideline recommendations in clinical practice. In spite of improved methodological process, wide endorsements, and dissemination of guidelines; MSK practice is still being fraught by limited accessibility, and applicability of guideline recommendations. This may be due to failings in the way they have been conceptualised for use, implemented and/or translated into real world practice.

Historically, and in response to several shortcomings, citizen science and models for public participation has led to maximizing public assets, competencies, and knowledge for improving health research and delivery in developed health systems [9]. Specifically, PPI in research have led to several advances in the field of MSK research e.g., the establishment of the Cochrane musculoskeletal consumer group, patient involvement with outcomes research and establishment of patient research partner groups [10]. However, inconsistencies in processes versus impact evaluation, failure to distinguish between PPI in research versus PPI in evidence-based knowledge mobilisation, and PPI in healthcare delivery, may have led to an oversight of the need for PPI in implementation and healthcare delivery.

For patients and careers who bear the health implications and socio-economic burden of living with MSK pain conditions, overcoming everyday challenges associated with MSK pain is an evolving task. Though evidence is always evolving, and guideline recommendations tend to be relatively stable over a period of time, yet, in reality, two days are guaranteed to be the same for MSK patients. Interpreting and applying guideline recommendations by people with lived experience is therefore an important consideration for implementation. Consequently, if the research-to-practice gap in MSK is to be closed, and evidence-based recommendations from guidelines successfully implemented to improve quality of care for MSK patients, a holistic approach to PPI is warranted. Such approach needs to be centred on true partnership throughout the continuum of evidence-based guideline production and implementation into practice, policy and service planning (i.e., patients as citizens and partners) [11, 12].

The aim of this article therefore is to explore and summarise PPI in evidence-based guidance implementation for MSK conditions. Beyond development and publication of evidence-based guidelines, we sought to map and examine PPI activities in guideline implementation, supporting adoption into practice and health care planning for people with MSK conditions.

Specific questions that guided our review were, across MSK conditions:

1. How have patients and public been involved with evidence-based guidance implementation activities beyond initial development, and dissemination of guidelines?
2. What strategies and contextual factors have enabled PPI in evidence-based guidance contextualisation and implementation?
3. What are the outcomes of PPI in guideline contextualisation and implementation on quality of care for MSK services and patients?
4. What are the current gaps in this field and what evidence is there in the literature regarding PPI contributions to MSK guideline implementation in LMICs?

## Methods

The review was guided by published methods for conducting scoping reviews [13] and the Scale for the Assessment of Narrative Review Articles [14].

### Search strategy and information sources

A search strategy using a combination of MeSH and free text terms from three categories i.e., musculoskeletal AND patient involvement AND guidelines/ implementation was developed to identify relevant publications in databases: MEDLINE, Embase and CINAHLPlus from their inception until July 28th, 2021 (see “Appendix 1”). No restrictions were applied for language or date of publication. In addition, searches (with keywords e.g., patient/public involvement, guideline implementation/adoption) of NICE, WHO and Guidelines International Network (G-IN) repositories were conducted to identify other relevant reports that may not have been profiled in bibliographic databases. References of relevant literature were hand-searched, and citation tracking of index reports and articles through google scholar were conducted to supplement database searches.

### Study selection

Eligible for consideration for this review were articles of any design reporting on PPI for the purpose of guideline contextualisation and/or implementation for any MSK condition in any health settings globally. We defined PPI in guidelines implementation as any activity involving patients, public contributors, and public partnerships to improve adoption, sustainment, and scale-up of evidence-based recommendations [15]. Such activities should not be limited to dissemination and language translations of guidelines only but may also include adaptation of guidelines to local or organisational contexts, training and use of evidence-based recommendations in clinical consultations, planning or commissioning of care [16]. However, brief commentaries of PPI in studies without specific application to evidence-based guideline implementation activities were excluded.

Study selection was managed using a systematic review software (COVIDENCE https://www.covidence.org/). Eligibility criteria were discussed and agreed prior to screening. Titles and abstracts were subsequently single screened using an inclusive approach—where there were uncertainties regarding eligibility, they were included for full text screening. On the other hand, full texts were double screened for eligibility independently by reviewers (OB & SD). Disagreements regarding eligibility were resolved by discussion. Eligibility criteria for included studies is presented in Box 1 below.

**Box 1:** Eligibility criteria

	Inclusion criteria	Exclusion criteria
Conditions	Population: Guideline related to adults, 18 years and older with any MSK conditions	Studies among paediatric populations Studies for other conditions or for which over 50% of patients were non-MSK
PPI participants	Studies reporting PPI recruitment, and involvement activities	Studies mentioning PPI but without any details of actual recruitment or PPI activities
Purpose (PPI) involvement	Guideline contextualisation to local/practice settings PPI in guideline implementation Consideration for health service planning/care organisation policy developments/ Monitoring and evaluation of guideline impact	Predominantly research Predominantly guideline development process (e.g., mention of PPI as part of “stakeholder consensus” at development stage) Articles evaluating the quality of guidelines with AGREE or any other instrument were not eligible
Outcomes of interest	Patient health related outcomes (e.g., Quality of life, shared decision making, acceptability) Sustained adoption and use of guidelines in practice Impact evaluation after guideline uptake (including impact on service delivery)	

The criteria are used to screen for eligible studies sequentially, in the following order:

MSK Conditions y/n; PPI participants y/n; Purpose of involvement y/n; Outcomes y/n;

A NO at any stage in the process leads to exclusion of the article

No restrictions on study design /settings or language

### Extraction of data

A data collection proforma designed and tested a priori (by reviewers with a sample article) was used to extract data including each study’s location (country) of PPI activity, aims, study design, methods, target settings for implementation of evidence-based recommendations, specific MSK conditions being addressed and records of PPI contributors and recruitment. Included articles were explored for critical information regarding the context for PPI, levels of PPI (based on adaptations of Bate and Robert’s [17] continuum of patient involvement) [17], outcomes/impact of such involvement and possible mechanisms for success of PPI in guidelines contextualisation and implementation. As the focus of this review was to provide an overview on the current state of evidence regarding PPI in guidelines implementation, articles fully satisfying our pre-defined eligibility criteria were only subjected to data extraction and not quality appraised [13]. Data were extracted by one reviewer using the customised data collection proforma and independently checked for consistency and completeness by a second reviewer. Where required, clarifications were sought and disagreements between reviewers (OB, SD) were resolved by discussion.

### Evidence synthesis

The narrative synthesis framework [18] and the continuum of patient involvement proposed by Bate and Robert [17] was used to guide synthesis. Firstly, the synthesis process involved tabulation**,** groupings, and classification of PPI involvement for implementation across included studies. Tabulated data were then interrogated independently by two authors (OB, SD) for patterns within the evidence base, exploring relationships (similarities and differences) and describing PPI implementation activities and outcomes between studies. Data were analysed to broadly address the first three questions, mainly to (i) identify and profile PPI activities in relation to the design, delivery, and evaluation of evidence-based guidance implementation; (ii) highlight strategies and contextual factors, particularly levels of PPI enabling evidence-based guidance implementation; and (iii) outcomes of PPI in guideline contextualisation and implementation on MSK services and patients. Outcomes of PPI were considered as either patient health related (e.g., quality of life, shared decision making, self-efficacy) or service-related (e.g., guideline uptake/adherence, informing policy or care commissioning). Groupings of PPI activities, contexts, and outcomes of PPI were validated in discussions among the review author team (OB, SD, OA, KD) and also with PPI co-authors (JB, LP). The robustness of the synthesis in line with tabulated evidence were reflected upon and discussed. Preliminary synthesis and review findings were further discussed and gaps in current evidence identified across the first three review questions were highlighted in review team meetings. Implications for further research and practice were then co-developed on the basis of highlighted gaps in evidence. One reviewer (OB) conducted an initial conceptual mapping of the data and created a visual representation of PPI in evidence-based guideline implementation process. These were further discussed among the author team, subsequent refinement led to the development of a conceptual framework for PPI in guideline implementation.

### Patient and public involvement and author team

Two members of Keele’s Lay Involvement in knowledge mobilisation (LINK) group contributed to and provided patient perspective to this review (JB, LP). The LINK group is made up of patient and public contributors who bring personal and volunteering networks and experiences from national charities, local community groups, patient support groups, and NHS organisations, to help support implementation activity, facilitate transfer of knowledge and innovations derived from research projects into real life practice. As PPI co-authors, JB and LP participated in meetings where PPI activities, processes, and guidelines implementation outcomes from included studies were discussed. JB and LP provided insights into what these findings might mean in real life, drafted PPI perspectives, and commented on draft manuscripts. LP also co-drafted the plain English summary of the review with OB (Additional file 1). Review authors have professional backgrounds in social science, evidence synthesis, applied health research, knowledge mobilisation, implementation science, physiotherapy, and general practice. All authors contributed to critical interpretation of study findings.

## Findings

### Characteristics of included studies

**Fig. 1:**
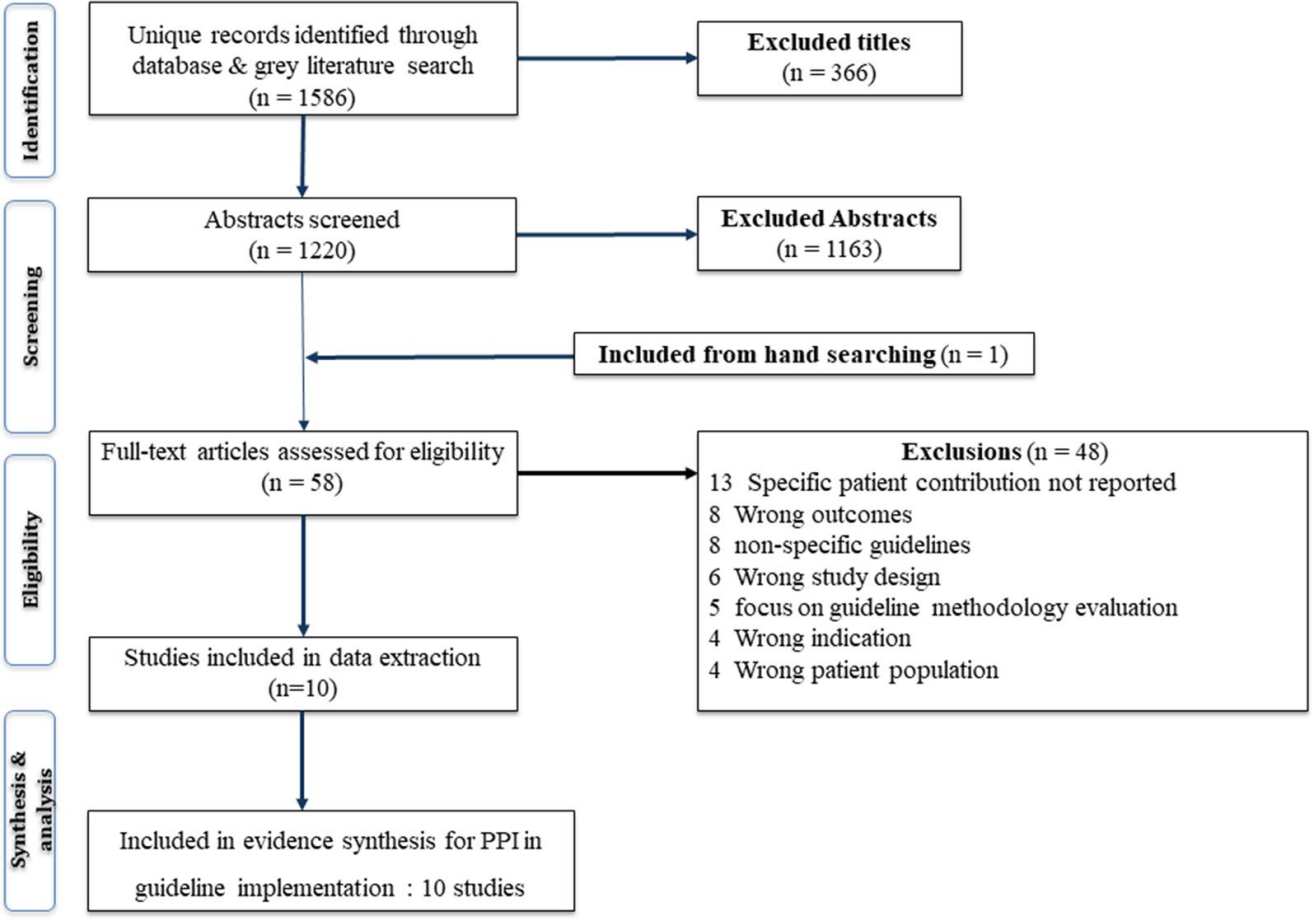
Flow chart of the review process

A total of 1586 titles and abstracts were screened as they potentially reported on PPI in the implementation (design, delivery, or evaluation) of evidence-based guidance for MSK conditions. Of these, 58 full texts were assessed for eligibility. Studies were excluded mostly because they did not report specific patient contribution apart from single statements that mentioned patients as part of stakeholder meeting(s); were reports of initial guideline development process (not implementation), related to non-specific guidelines or non-specific musculoskeletal condition (i.e., general patient involvement) or were related to guideline methodology evaluation. A summary of the review process outlining study selection is presented in Fig. 1.

Our final sample of studies included ten articles [19–30] published between 2009 and 2020 [27, 30] and involving patients and public, researchers and healthcare professionals in evidence-based guidance implementation processes. Three studies [19, 23–25, 30] (all related) specified the profile of public contributors involving: commissioners of care, healthcare managers, and public administrators in their implementation activities. Activities relating to PPI in the implementation evidence-based guidance for MSK conditions originated in Europe, most deriving from the UK and involving other European counties (Netherlands, Norway, Denmark, Portugal, Belgium, Cyprus, Czech Republic, Ireland, and Romania—n = 8, some studies were multi-sites) [19–26, 28, 30], with 1 from Asia (Turkey) [29], and 1 from Africa (South Africa) [27]. All were qualitative in design, but two were mixed methods studies (including consensus methods, interviews and focus groups from a nested cluster randomised controlled trials) [25, 30]. MSK conditions for which studies reported PPI in evidence-based guidance implementation were osteoarthritis (OA) [19, 20, 23–25, 30], rheumatoid arthritis [21, 22, 29], ankylosing spondylitis [26], chronic musculoskeletal pain [27] and psoriatic arthritis [28]. All included studies involved PPI contributors who had lived experiences of the MSK conditions (Table 1).

**Table 1:** Characteristics of included studies

First Author /Yr.	Country (target/conduct of PPI activity)	Brief study aim(s)	Study design	MSK Condition(s)	Total sample of PPIE participants if specified	Relevant Healthcare settings if applicable	Target for PPIE activity related outputs	Additional notes/General comments on study/population characteristics
Blackburn 2017 [19]	UK, Netherlands, Norway, Denmark and Portugal	1. Simultaneous support and PPI involvement on OA guidelines implementation. 2. Quality indicators as part of OA guideline implementation.	Qualitative	Osteoarthritis	7	Primary care	Patients and researchers	Describes overarching PPI involvement, process of PPI support and steering of associated implementation projects
Campbell 2018 [20]	UK, Netherlands, Norway, Denmark and Portugal	To support translation and cultural adaptations of the OA Guidebook (based on NICE OA guidelines) appropriate for local context and use by patient champions and health professionals	Qualitative	Osteoarthritis	15	Varied-main target is primary care	Patients & HCPs	15 patients formed a CoP who also engaged with various OA patient organisations
DeKeyser 2015 [21]	Belgium	To develop patients’ version of the EULAR recommendations and enhance the level of information available to increase possibilities of self-management	Qualitative	Rheumatic and Musculoskeletal diseases	18	NR	Patients	Involved patient partners trained to be partners in research. Contributors previously trained and experienced in PPI activities
De Wit 2011 [22]	Cyprus, Czech Republic, Denmark, Ireland, Norway, Portugal, Netherlands, Romania and UK	To develop a patient version of the Treat to target (EULAR recommendations for Rheumatoid arthritis).	Qualitative	Rheumatoid arthritis	9- aged 31–66yrs, 1 male	NR	Patients	Recruitment and selection of participants was aided by a large patient organisation: EULAR Standing Committee of Patients with Arthritis/Rheumatism in Europe through purposive sampling accounting for geographical variation, gender and age. English language proficiency (read/speak) was required.
Dziedzic et al. 2018, 2014 [23, 24], Blackburn 2016 [25]	UK	Study investigated approach to implementing core NICE OA recommendations in primary care supported by PPI	Mixed methods study (including consensus methods) with a nested cluster randomised controlled trial	Osteoarthritis	10: 5 males, 5 females; aged 52–80 years	Primary care	Patients & HCPs	Newly formed dedicated Research user group with OA worked in partnership with researchers throughout the study, including the development of patient reported Quality indicators for evaluating use of guideline recommendations in primary care OA consultations.
Kiltz 2010 [26]	Germany	To describe how the English lay version of EULAR recommendations was translated into German by a group of patients.	Qualitative (evaluation)	Ankylosing Spondylitis	13 patients from German Language area distributed (10 AS patients from Germany, 2 from Switzerland and 1 from Austria).		Patients	PPI contributors were also asked to confirm their acceptance of the German translation and degree of consent to the content of the recommendations.
McCaul 2020 [27]	South Africa	To provide contextually relevant, evidence-informed guidance on the assessment and management of chronic musculoskeletal pain (CMSP).	Qualitative (case studies- only 1 of 4 presented here was relevant to MSK)	Chronic musculoskeletal pain	Sample not reported. Patient input was sought as part of development, along with broader stakeholderconsultation	Primary care	HCPs	Single study from Africa. Details of actual PPI activities/process of involvement and demographics of contributors not reported.
Özgöçmen 2009 [29]	Turkey	To evaluate the Turkish translation of the patient version of the (ASAS) and (EULAR) recommendations for the management of ankylosing spondylitis.	Qualitative	Ankylosing Spondylitis	12 patients (4 female, 8 males, mean age 39.5 and disease duration 11.5 years from various provinces of Turkey).		Patients	PPI contributors were members of the Ankylosing Spondylitis Patient Society of Turkey.
O'Sullivan 2017 [28]	UK	To describes how a patient-oriented guide to treatment recommendations was developed by GRAPPA’s patient research partners.	Qualitative	Psoriatic Arthritis	3 members (lead writer and 2 co-writers) volunteered to prepare an initial workingdraft of the guide.	Primary care	Patients	Unclear whether PPI contributors involved or not. Streamlined project team reported experience in writing, editing, graphic design, and project delivery skills yielded a better quality first draft within a reasonable time period. Patient research partners appear to be highly trained and well experienced in research related to the (PRP).Addressed readability and accessibility to broad patient audience.
Swaithes 2020 [30]	UK	To understand uptake of OA recommendations, and explore the journey from a clinical trial to implementation	Qualitative–secondary analysis of focus groups and stakeholder interviews	Osteoarthritis	Not specified but sourced from a large network of public contributors and managers involved with healthcare	Primary care	Researchers & Commissioners to inform implementation	Linked to NICE OA guideline implementation projects

*PPI* Patient and Public Involvement; *PPIE* Patient and Public Involvement and Engagement; *CoP* Communities of Practices; *HCPs* Health Care Professionals; *OA* Osteoarthritis; *NICE* National Institute for Clinical Excellence; *CMSP* Chronic Musculoskeletal Pain; *ASAS* Assessment in Spondylarthritis International Society; *AS* Ankylosing Spondylitis; *EULAR* European League Against Rheumatism; *PRP* Patient Research Partner; *GRAPPA* Group for Research and Assessment of Psoriasis and Psoriatic Arthritis

### Review objective 1: PPI activities in evidence-based guidance implementation

PPI activities were nested within both design and delivery [19, 28], or delivery only [20–22, 26, 27, 29] phases of guidance implementation. Two studies (both related) [23–25, 30] embedded PPI activity inclusive of design, delivery, and evaluation phases of guideline implementation. PPI activities involved patient contributors in user panels or advisory meetings for: (i) steering associated evidence-based guidance implementation projects, (ii) planning evaluation of guidelines implementation, (iii) language translation, (iv) development of patient version of recommendations, and (v) cultural adaptations and contextualisation of original version of guidance and recommendations.

As successful implementation of evidence-based guidance into practice often requires dissemination as a key step, unsurprisingly, many of the PPI activities reported were related to guideline dissemination and development of guideline dissemination products. Intended target audience for MSK guidelines dissemination products for which PPI related involvement were reported were mostly patients themselves [21, 22, 26, 28, 29]. For many of the PPI language translation activities, high-level agreement on content, acceptability, and accessibility of MSK guideline dissemination products were often reported between PPI contributors and healthcare professionals (HCPs) who took part [22, 26, 28, 29]. Two projects [20, 23–25] adopted a more creative stance, targeting resources for dual use by patients and healthcare providers in primary care and community settings.

### Review objective 2: Levels of patient and public involvement

More than half of the articles (n = 6: Involvement process n = 2, Consultation, n = 4) included consultative activities typical of low-level involvement (i.e., where depth of involvement was not spelt out in detail, was difficult to unpick or simply required patients input at late stages of implementation activities (e.g., one day meeting/conferences to suggest wordings or vote agreement to previously developed implementation products. Other four articles (3 of these concerned related projects) demonstrated higher-level involvement with PPI (i.e., Shared partnership and leadership n = 4). These often engage patient and public contributors in co-design (including planning, deliberation, reflective processes) where PPI worked together with researchers/HCPs to create solutions for mobilising knowledge and were actively involved in steering the planning, delivery, and evaluation of implementation activities (Table 2).

**Table 2:** Contexts, possible mechanisms and outcomes of PPIE in implementation of evidence-based guidance for MSK conditions

First Author /Year of publication	PPIE activities	Context for PPIE involvement	Levels of PPIE	Outcomes of PPIE involvement	Probable mechanisms for effectiveness of PPIE in contextualisation and implementation	Additional notes/ Other relevant findings
Blackburn 2017 [19]	Design/planning: 1. steering guideline implementation project 2. evaluation of guideline implementation	Implementation in clinical practice Guideline monitoring/quality improvement, and implementation for shared decision making, patient education and empowerment	Shared partnership and leadership	Patient Health outcomes: NR Empowerment/enablement/self-efficacy: NR Guideline uptake/adherence: NR healthcare organisation/practical issues: international collaboration of PPIE within implementation projects	**Contextualisation** 1. Patient Champion as part of guideline implementation project steering committee 2. PPIE support alongside involvement e.g., in development of a set of glossary of terms to support the involvement of patient panel members throughout the project **Implementation** Emphasis not only on language translation but also cultural adaptation of patient information resources	Abstract only- lacking actual details and description of PPI in every stage An example of PPI in planning guideline implementation strategy Reports consideration for factors that may affect context such as settings, views of target users and some shared learnings with relevant health care organisation
Campbell 2018 [20]	Delivery phase: cultural adaptations and contextualisation of a lay version of OA guidance and recommendations	Implementation for shared decision making/patient education/empowerment implementation in clinical practice	Shared partnership and leadership	Patient Health outcomes: NR Empowerment/enablement/self-efficacy: feasibility and effectiveness of patient CoPs Guideline uptake/adherence: NR Healthcare organisation/Practical issues: Implementation of OA guidelines—The production and dissemination of a new resource: culturally adapted, consistent and accurate patient information booklet to aid clinical practice and consequently patient outcomes	**Contextualisation** Patient voice in language, images, content **Implementation** 1. PPIE leadership and ownership through CoPs and wider engagement with local patient organisations. 2. Wider engagement with other stakeholders could have enhanced uptake and implementation in practice. 3. Cultural adaptations and considerations for how local health systems works. Nb: output was targeted and localised to the different health systems in the countries involved	Elements of successful PPIE: consistency check with national guidelines; shared learning across countries; freedom of each CoP to adopt a process appropriate to their specific context Offers opportunity for PPI to challenge and evaluate Includes drive to scale up and share learnings around guideline implementation
De Keyser 2015 [21]	Delivery phase: development of patient version	Implementation for shared decision making/patient education/empowerment	Involvement (process)	Patient Health outcomes: NR Empowerment/enablement/self-efficacy: NR Guideline uptake/adherence: NR healthcare organisation/Practical issues: NR	**Contextualisation** 1. Training of PPIE participants and partners to ascertain understanding and familiarity with original EULAR recommendations 2. Collaboration with healthcare professionals to guarantee quality and ensure translations are a correct reflection of the original documents **Implementation** Available resources such as: Link with EULAR, expert academics and researchers?	Abstract only- lacking actual details and description of PPI in every stage Possible link to development of guideline implementation strategy
De Wit 2011 [22]	Delivery phase: development of patient version	Implementation for shared decision making/patient education/empowerment	Involvement (process)	Patient Health outcomes: NR Empowerment/enablement/self-efficacy: NR **Guideline uptake/adherence:** An easy tool to facilitate uptake of T2T recommendations in practice (among HCPs) **healthcare organisation/Practical issues:** enhance shared understanding and ensure smooth processes organisation of RA treatment and monitoring according to recommendations. Outcome of current process: “Participants noticed that the T2T recommendations, like the EULAR/ASAS recommendations, have a strong focus on body functions and structures, while patient-centred care in rheumatology also requires, besides medical expertise and monitoring, non-pharmacological and psychosocial support”	Contextualisation PPIE involvement had been preceded by pre-work among a core group:—four members of the international T2T Steering Group, including one patient representative), produced a draft version of the T2T recommendations in lay language which was discussed, amended and reworded during a 1-day consensus meeting with nine RA patients and moderated by two members of the core group (a patient and researcher). Also, 5 of 9 participants had been previously involved in the consensus meeting leading to the development of T2T recommendations.—Continuity or overfamiliarity with content affect output? Implementation glossary of terms in lay language was also developed to accompany patient version recommendations	Product developed by experienced patient representatives fluent in English. No report of validation among lay patients. Translation into different languages, testing, and processes for dissemination in different countries were agreed as subsequent next steps study described details of PPI participants recruitment and selection as well as detailed level/process of involvement. Missing detail on development stage highlighted during contextualisation Examples of scale up and shared learnings but may have missed opportunity for PPI contributions to define and confirm what implementation should be
Dziedzic et al. 2018, 2014 [23, 24], Blackburn 2016 [25]	Design, delivery, and evaluation	Implementation in clinical practice Also implementation for shared decision making/patient education/empowerment; Reference to another quality indicator (clinician/research led) in Norway as a basis for comparison and content validity	Shared partnership and leadership	Patient Health outcomes: There were no statistically significant differences in SF-12 PCS: mean difference at the 6-month primary endpoint was − 0.37 (95% CI − 2.32, 1.57) **Empowerment/enablement/self-efficacy:** improvement in patient enablement suggests a beneficial effect of the intervention on the capacity of patients for self-management—one of the targets of NICE core guidance **Guideline uptake/adherence:** Uptake of core NICE recommendations by 6 months was statistically significantly higher in the intervention arm compared with control: e.g., increased written exercise information, 20.5% (7.9, 28.3) **healthcare organisation/Practical issues:** Identifying important and relevant quality indicators of OA in primary care consultations from a patient’s perspective. The OA QI (UK) was developed to assess the uptake of treatment recommended by NICE and complements the new NICE Quality Standards of Care for OA. The development of two OA indicator questionnaires (quality indicators validated for Norwegian OA and UK consultations) coincidental but led to further research to compare patient reported OA QIs across European countries	**Contextualisation** research team met with RUG members to co-produce the OA QI (UK) questionnaire. Discussion meetings were facilitated by the Centre’s PPI Support Worker/Coordinator, the MOSAICS study Chief Investigator and a trial coordinator. The PPI Support Worker/Coordinator provided a key role by attend the meetings with RUG members to provide assistance and support, prior, during and after meetings. Discussion notes from the meetings were recorded on flip charts and in meeting minutes. Following each meeting, a summary of the outcomes and decisions written in plain English was sent to the RUG members to acknowledge their contribution and verify that all views had been captured. RUG members were also given the opportunity for further comment at the start of the next meeting **Implementation** The discussion groups took place over a three-year period from 2009–2012. extended gaps between meetings regarding the OA QI (UK) development, the timings of the meetings were governed by the study timeline. However, RUG members were provided with feedback of the meeting and given the opportunity to comment. This process built upon existing working relationships and trust between the RUG and researchers	NB: RUG membership was not greatly diverse, in terms of age, ethnicity, and physical abilities. While obtaining a range of perspectives is the objective of PPI in research and not necessarily ‘representativeness’, it is possible however that the OA QI (UK) does not cover the full range of quality indicators relevant to the population of patients with OA. Nevertheless, the sequential and iterative development of the OA QI (UK) allowed the researchers and RUG members to review and critique earlier suggestions made by the RUG Targeted approach to guideline implementation. Strategy developed close to guideline development though not by the development group. PPI contribution along the continuum included contextualisation, evaluation, refining, scale up and shared learnings
Kiltz 2010 [26]	Delivery phase: Translation and brief validity of translations	Guideline impact evaluation	Shared partnership and leadership	Patient Health outcomes: NR Empowerment/enablement/self-efficacy: NR Guideline uptake/adherence: NR healthcare organisation/Practical issues: NR	**Contextualisation** Patients discussed language, content and evaluated proposed recommendations Implementation NR	Limited detail but article presents a case of PPI in scale up of guideline implementation products The report may also have missed opportunity to capture PPI contributions in defining the specific implementation strategy
McCaul 2020 [27]	Delivery phase: cultural adaptations, contextualisation of guideline recommendations	Guideline adaptation and contextualisation in a resource-constrained setting	Consultation	Patient Health outcomes: NR **Empowerment/enablement/self-efficacy:** **Guideline uptake/adherence: NR** **healthcare organisation/Practical issues:** access to funding and dedicated human resources were a significant challenge to adapting contextualised recommendations in intended setting	**Contextualisation** Stakeholders evaluated proposed recommendations **Implementation** An end-user document with an implementation plan is currently being developed	Key learnings revolved around navigating funding and human resource challenges, whereas opportunities include addressing guideline training gaps and investing in strengthening adaptation and contextualisation of guideline recommendations through stakeholder engagement for efficient guideline development and enhanced uptake PPI contributions indistinct though involvement was aimed at addressing a mix of service delivery (care pathway) and clinical content too Impact of PPI on guideline contextualisation could not be assessed. Missed opportunity for PPI contributions to define and confirm what implementation should be
Özgöçmen 2009 [29]	Delivery phase: Translation and patient evaluation	Guideline impact evaluation	Involvement (process)	Patient Health outcomes: NR **Empowerment/enablement/self-efficacy: NR** **Guideline uptake/adherence: NR** **healthcare organisation/Practical issues:** possible changes in the applications of drug recommendations were referenced from a linked study due to differences in the legislation and reimbursement institutions between European countries	**Contextualisation** Patients discussed language, content and evaluated proposed recommendations **Implementation NR**	PPI centred at latter end for scaling up guideline dissemination product
O'Sullivan 2017 [28]	Design and delivery-phases	Guidelines development	Consultation	Patient Health outcomes: NR Empowerment/enablement/self-efficacy: NR Guideline uptake/adherence: NR healthcare organisation/Practical issues: NR	**Contextualisation** Patients involved in development of guidance but unclear how and to what extent **Implementation NR**	The project team used a professional graphic designer to help with the graphic and formatting elements of the project but found this stage demanding and time-consuming? Challenges with processes and supporting PPI were highlighted Patient voice indistinct. PPI contributions were targeted at later end for scaling up guideline dissemination product Impact of PPI on guideline contextualisation could not be assessed
Swaithes 2020 [30]	Design/planning: input into design and interpretation of findings	Implementation in clinical practice	Involvement (process)	Aided formative evaluation and capturing mechanisms involved in implementation of guideline recommendations	**Contextualisation** **NA** **Implementation** Expertise and lived experience maximised to inform formative evaluation and capture nuances and context-based factors influencing OA guideline implementation	Focussed PPI input into capturing implementation processes and future learning. Public contributors were part of an established and experienced group for lay involvement in knowledge mobilisation Refining and evaluating PPI in guideline implementation

*PPI* Patient and Public Involvement, *PPIE* Patient and Public Involvement and Engagement, *CoP* Communities of Practice, *OA* Osteoarthritis, *NICE* National Institute for Clinical Excellence, *ASAS* Assessment in Spondyloarthritis International Society, *EULAR* European League Against Rheumatism, *RA* Rheumatoid Arthritis, *HCPs* Health Care Professionals, *RUG* Research User Group, *QI* Quality Indicator, *T2T* Treat to target

PPI efforts were mostly (n = 9 studies) targeted at primary health care settings. No study formally evaluated or reported patient and public experiences of the process of being involved in evidence-based guidance implementation.

### Contextual factors for PPI in evidence-based guidance implementation

Context for PPI activities as part of evidence-based guidance implementation across the studies included (i) support of well-established/funded organisations, (ii) patient leadership and involvement in implementation planning /design phase, and (iii) country, culture, and training. Except for the one study from Africa, included studies worked on implementation of recommendations that were developed or supported by well-established organisations (e.g., EULAR- 4 studies, NICE/NIHR -3 studies, and the Group for research and assessment of psoriasis and psoriatic arthritis (GRAPPA -1 study). Links to these organisations aided funding, recruitment and selection of PPI contributors, access to a wide pool of patient research partners often with previous experience of PPI in research (“patient research partners”), as well as extended networks and avenues for further dissemination and implementation activities. Patient involvement activities in such studies also followed similar process of conduct and reporting [21, 22, 26, 29].

An important example of the influence of patient leadership and involvement in implementation planning /design phase can be seen in the study by Campbell 2018 where patient and public contributors involved in the implementation activities subsequently formed a “Community of Practice” and then started to engage with other networks of OA patient organisations across all the European countries involved [20]. This demonstrated continuity of PPI in implementation where newly formed OA research user groups were able to work in partnership with researchers throughout a five-year programme of implementation and research. In this review, this was the only reported example of guideline implementation evaluation planned a priori and nested within implementation delivery with active patient involvement.

### Review objective 3: Outcomes of PPI in MSK evidence-based guidance implementation

In terms of patient health related outcomes (i.e., Quality of life, shared decision making), only one study [23–25] carried out post implementation evaluation to report patient health related outcome following PPI in evidence-based guidance implementation. A process which had earlier resulted in the development of a set of quality indicators of primary care consultations for OA from a patient’s perspective. The study however reports no statistically significant differences in quality of life of patients (including those who participated in “model consultations” and those who did not) as assessed using SF-12 PCS: mean difference at the 6-month primary endpoint was − 0.37 (95% CI − 2.32, 1.57).

There was no direct evidence, or reports of sustained adoption and use of guidelines in practice across most of the included studies beyond short term PPI involvement in implementation activities. In relation to impact on service delivery, one study [23–25] led to PPI supported OA quality indictor (patient’s perspective) complementing the NICE Quality Standards of Care for OA that were well received/used in practice and was later conceptualised for use in another care setting (Norway). There were no further organisational or service-related outcomes reported across studies.

### Review objective 4: Review highlights and current gaps in literature

Low-level PPI involvement limited to basic involvement and consultative activities relating guideline dissemination products mainly, highlight a significant knowledge and implementation gap for MSK guidelines and evidence-based recommendations. This was also evident in LMICs (based on a single report from South Africa) with limitations and uncertainties around actual PPI contributions [16]. Many reports lacked information about recruitment and demographics of PPI contributors. PPI activities were not included in the guideline implementation design phase, neither was there evidence of equal partnership and stake in the consultative activities.

Based on currently, available literature, guideline uptake strategies appear to be focussed on dissemination and initial acceptance and may have resulted in limited evidence of sustained use, and adherence. Little is known about optimal implementation strategy by which sustained use can be achieved for improving care and minimising variations in practice.

From this review, the level of PPI in implementation work reflects the level of training, country specific over-representation (specifically the UK) and cultural influences on practice in different care settings. Training, development, and practice of PPI in implementation has not spread much beyond Europe- though it is possible that these activities may be occurring at low levels in some form but are not yet well reported in literature. This is important for future reporting so that guideline implementation activities and PPI involvement within these can be rightly accrued.

Eligible studies contributing to this review have all been published over the last twelve years (2009–2021). Though our search strategy was not restricted by date, findings show that in recent times, there has been an increase in the amount of lay and public versions of similar evidence-based recommendations being produced for use in different settings for different audiences. Conceptual understanding of guideline recommendations from such versions may differ for different audiences.

Our PPI co-authors considered the need to address practicalities of applying lay versions of guideline recommendations in real life with PPI support as a necessary next step in MSK guideline implementation. In addition, the PPI co-authors also expressed concerns that discordance between HCP-patient beliefs, different expectations about what the outcome of MSK consultations should be can jeopardise shared decision-making, guideline uptake and adherence. Therefore, an important focus for future implementation research for MSK conditions should involve a proactive, a priori plan for guidelines dissemination products that could be targeted for use by both lay and professional end-users. The G-IN toolkit is an example of such an initiative but has limited uptake in MSK field. Remarkably, the recently updated G-IN public toolkit (https://g-i-n.net/toolkit/) [31] illustrate case studies of PPI in guideline implementation (including shared learning from a new rheumatoid arthritis guideline implementation) [32], and also includes practical advice for PPI in guideline activities. However, the G-IN toolkit and currently lacks reference and applications to guideline contextualisation and implementation in LMICs.

In addition to a palpable knowledge gap relevant to PPI in evidence-based guideline implementation, lack of skills, cultural influences such as paternalism in healthcare settings may also contribute to the limited evidence for patient and public partnership in evidence-base guidance implementation for MSK conditions in LMICs. Increased funding and deliberate engagement, greater international collaboration, implementation research and trusts are needed to build capacity, collaboratively improve knowledge base, and partnerships for PPI in MSK guidelines implementation.

Irrespective of world region, there was an obvious lack of reported PPI activities in concurrent design, delivery and evaluation phases of guidelines implementation found in this review. Guideline producing organisations in collaboration with stakeholders should prioritise implementation design, delivery and evaluation that is ideally developed in parallel with the evidence-based guidance recommendations and not in isolation.

### PPI author perspectives on current evidence and way forward

In response to funding requirements and patient advocacy initiatives, public contributors are increasingly invited to contribute to MSK research (e.g., grant applications, research reporting purposes). As a result, PPI in MSK research is more common for seeking opinion about what is ‘doable’ at the beginning of research cycle but without contributors hearing of when research (to which they contributed) has been incorporated into MSK guidelines. Patients who have contributed to research processes are often not aware of MSK guideline findings. There should be a process for linkage and continuity.

Notably, PPI has established relevance in issues relating to health literacy, translation activities and acceptability of the language or text used in guideline dissemination products but not so much about the actual practicalities of applying these recommendations in real life practice alongside HCPs. PPI in implementation and knowledge mobilisation should not be stopping short at producing materials. Community involvement should continue with implementation using new and existing links that were already created through PPI with research. Continuity from research through to implementation should be guaranteed with funding for implementation planned and ready subject to review, as we know that things evolve. Challenges also remain with limited distribution and awareness of guidance-based products and how best and when to use them. Full involvement of PPI from research to guideline recommendations and implementation is important for improving quality of care for MSK patients.

### A conceptual framework for PPI in contextualising and implementing evidence-based guidance in practice

**Fig. 2:**
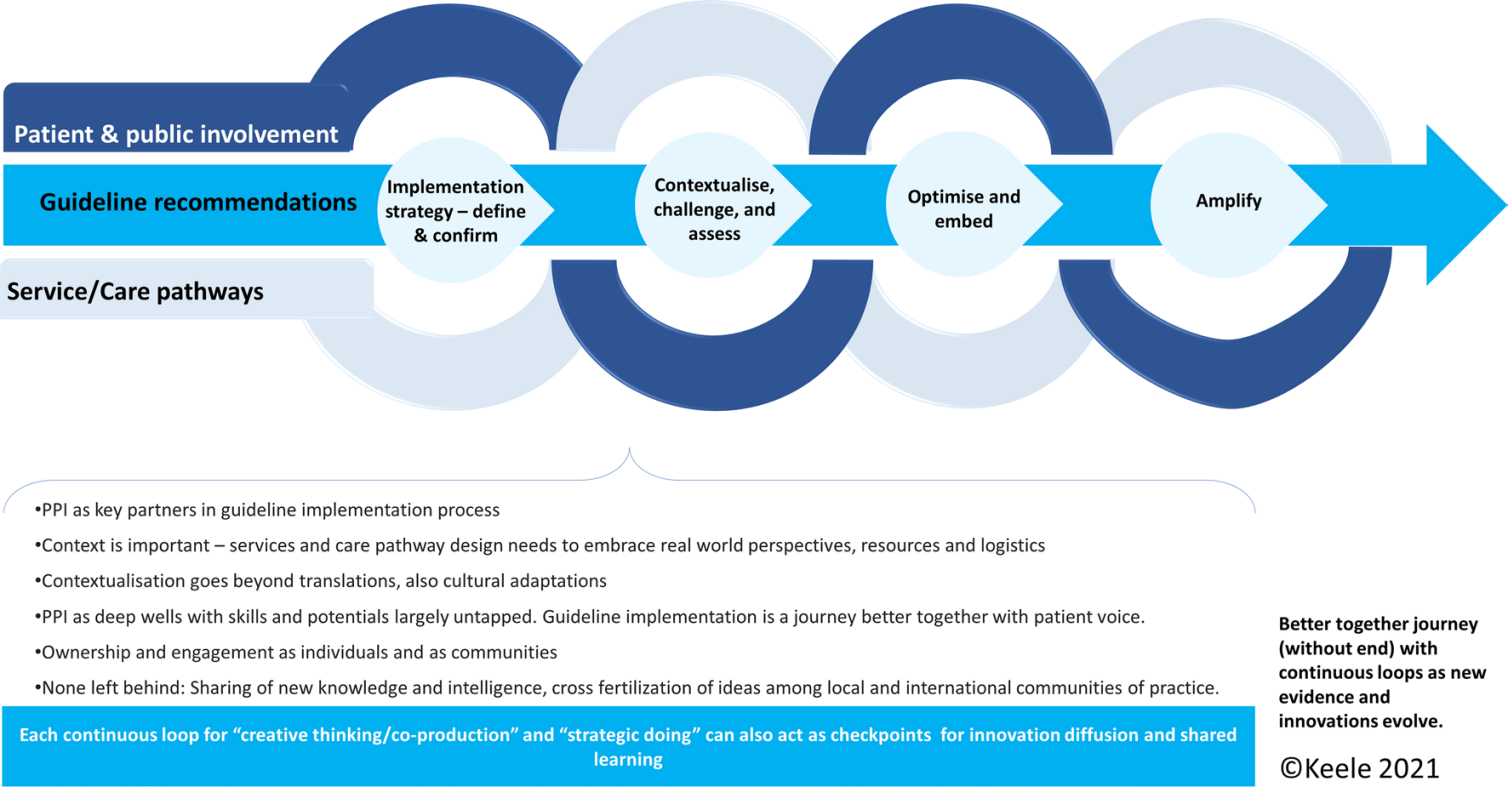
The Alliance Framework for conceptualising PPI in guideline implementation

PPI activity and evaluation has long been a subject of discussion for research and is an important issue to address in implementation. There is currently no framework for conceptualising PPI contributions to guideline implementation activities. The team (with experience of PPI, knowledge mobilisation/implementation, and MSK research) used evidence from this review (Table 3) and expertise gained in the practical application of theory to explore key principles and consideration for PPI in evidence-based guidelines implementation in an “ideal world”. In doing so, we conceptualised a continuous loop of “creative thinking/co-production” and “strategic doing” with PPI as new evidence evolves and is contextually translated into practice. We propose the “Alliance” framework (illustrated in Fig. 2) with the aim to underscore the need to:

1. define and confirm with PPI, guideline implementation strategy at development stage,
2. contextualise, challenge, and assess real world impacts and implications of guideline recommendations with PPI
3. optimise as needed and embed the use of recommendations in service designs, through coproduction
4. further amplify innovations through peer to peer, community-based and systems wide advocacy.

**Table 3:** Mapped PPI activities across implementation process—development of conceptual framework for PPI in guidelines implementation

First Author /Year of publication	PPIE activities	Elements of implementation exemplified	Targets of PPI outcomes	Notes
Blackburn 2017 [19]	1. Participated as panel members for planning and steering implementation project across 5 European countries 2. PPI (at country levels) co-developed language translation and cultural adaptation of OA guidebook for patients 3. Patient Panel members helped refine an OA Quality Indicator questionnaire (to be used for evaluating OA consultations in line with guideline recommendations). 2 for use in JIGSAW-E plan for evaluation of PPIE involvement and guardiance implementation	Implementation strategy—define & confirm Contextualise, and assess Optimise (through language translations) and embed in practice (patient champions) Amplify- international collaboration	Primary care, service/care pathway	Limited reporting of the details of PPI process
Campbell 2018 [20]	Patient and Public "CoP"- Community of practice established to: 1) review OA Guidebook and existing written patient information; 2) support language translation; 3) cultural adaptation: review of content, images and layout; 4) consistency check with national guidelines; 5) shared learning across countries	Implementation strategy—define & confirm through CoP approach Contextualise—cultural adaptations, images and layout to suit intended audience Optimise through content check with established guidelines and international collaboration	Primary care/community (including charity & patient advocacy groups)	Process was through discussions in meetings, reviewing and commenting on drafts
De Keyser 2015 [21]	PPI helped to: extract patient relevant information for self-management from EULAR recommendations; 2. Language translations; 3. disseminate to patient communities	Contextualise and optimise through—development of patient version Amplify through supported dissemination among patient community	Primary care/community	Limited reporting of the details of PPI process
De Wit 2011 [22]	PPI helped to: develop patient version of previously prioritised set of recommendations	Contextualise and optimise through—Formulating a patient version in lay language	Primary care/community	Consensus process used. Well established methodology. However, possible negative impact on contextualisation is unknown as PPI contributors were not allowed to make any changes in the content or meaning of the recommendations
Dziedzic et al. 2018, 2014 [23, 24], Blackburn 2016 [25]	PPI helped to 1. identify important and relevant quality indicators (QI) for patients with OA when consulting in primary care, 2. developed wording and response options for a self-report OA QI. 3. assessed (via comparison) content of the OA QI (UK) questionnaire with a parallel questionnaire developed in Norway. 4. co-developed training for HCPs on guideline-based OA consultation and the use of OA QI	Implementation strategy—define & confirm design, Contextualise, challenge, and assess Optimise and embed Amplify, scale up	Primary care	Methods used for PPI included facilitated discussions, co-productions, consensus process, review of drafts
Kiltz 2010 [26]	PPI helped to develop language translation and brief validity of patient versions	Optimise (through language translation) and contextualise to local settings: mainly targeted at patients	Primary care/community	
McCaul 2020 [27]	Cultural adaptations and contextualisation of actual guidance and recommendations	Contextualise to local settings: mainly targeted at health care professionals	Primary care, service/care pathway	Limited reporting of the details of PPI contributions and process apart from involvement on a consensus study alongside research experts and HCPs
Özgöçmen 2009 [29]	PPI activities mainly language translation and evaluation of content for accessibility	Optimise through translation and contextualisation to local settings: mainly targeted at patients	Primary care/community	
O'Sullivan 2017 [28]	PPI helped to: 1. establish need for and define strategy for implementation 2. draft patient relevant information/version of guidelines; 2. contextualise for use among target audience 3. planned dissemination to patient communities	Implementation strategy—co-defined needs and plans Contextualise and optimise through—development of patient version Amplify through supported dissemination among patient community	Primary care/community	Highlights PPI skills (including witting experience, editing, graphic design, and project delivery) and contributions (multiple drafts and discussions) to implementation project
Swaithes 2020 [30]	PPIE liaison in general practices	Implementation strategy—define Contextualise, and assess Optimise and embed in practice through PPIE liaison in primary care	Primary care, service/care pathway	Facilitated discussion, PPI viewpoints, process evaluation

The Alliance framework comprises of four-continuous loops that indicate:

- PPI as equal partners in guideline implementation process (not just in the development process). PPI voice and investment at every stage needs to be distinct and amplified.
- Context is important—services and care pathway design need to embrace real world perspectives, diversities of use, health systems, resources, and practicalities. PPI can help to factor context in. Guideline implementation is a journey that is better together with patient and public insights.
- Guideline contextualisation and dissemination for use by the public and HCPs goes beyond language translations, it also involves cultural adaptations. PPI can help to shape and facilitate this through community engagements.
- PPI can promote ownership of and engagement with service/care pathway improvement by individuals and communities.

This new framework complements known initiatives by the NICE patient experiences in guidelines and the PARE (People with Arthritis and Rheumatism) networks in EULAR to illustrate how PPI can influence interactions between research, policy and healthcare practice, and benefit diverse stakeholders. As it stands, the Alliance framework requires further input for development and validation. It is therefore being proposed in this first instance as a conceptual framework to further identify opportunities for PPI in care pathway development and also explore the need to increase diversity in PPI, sharing of new knowledge and intelligence across different health systems, and cross fertilization of ideas among local and international communities of practice.

## Discussion

We conducted a review of PPI activities in evidence-based MSK guidance implementation, explored strategies and contextual factors that may have enabled PPI in evidence-based guidance contextualisation and implementation, as well as current gaps in literature. A prevalent consultative activity with low-level PPI was found in current literature on implementation of MSK guideline recommendations. For LMICs, the gap in published evidence was found to be wider than envisaged.

A common strategy for evidence-based guidance implementation was translation into different languages and producing lay versions with the intent that culturally adapted, consistent and accurate patient information might enable patient informed decisions about treatment; and to facilitate patient-professional dialogue/shared decision-making process. However, these assumptions are yet to be backed up by strong evidence due to a lack of robust evaluation of implementation and observed low levels of guideline uptake and adherence. Similar to the wider literature on PPI in research, findings from this review shows the lack of evidence for any comprehensive approach on how to translate guidelines into practice. Our findings highlight the need for research that evaluate different implementation strategies in a local context, and the need for future implementation agenda to include understanding of the true impact, costs and possible drawbacks of PPI on implementation processes and outcomes.

Other important roles for PPI activities in evidence-based guidance implementation are largely missing or not visibly reported in current literature. This includes high level PPI and engagement in commissioning of care, and health policies. Our finding of limited PPI in healthcare implementation for MSK is in line with previous literature [5]. In their scoping review of reviews (though not specific to MSK), Modigh et al. [5] found a larger number of studies reporting PPI in research in comparison to healthcare and implementation. According to Forbat and colleagues four models of involvement [33], current advancement in PPI for MSK care is overtly concentrated on one end of the spectrum involving patients and public as consumer (with choice to purchase service). Our conceptual framework (“Alliance”) improves on this by conceptualising PPI in guideline implementation as an unending journey where PPI, and evolving evidence-based recommendations from guidelines can be innovatively integrated into service care pathways for better health outcomes. As such advances in PPI visibility in healthcare planning and policy may be important implementation next steps for MSK care.

An overwhelming gap for evidence-based guideline implementation and patient and public partnerships exists in LMICs. For instance, key initiatives to develop an international practice and research agenda on PPI in clinical guideline lacked specific involvement nor included focus on LMICs [34]. Given that research funding, dedicated human resources, and infrastructures to support new culturally sensitive clinical practice guidelines remains a significant challenge, guideline contextualisation and adaptation becomes one of the most viable opportunities for health systems strengthening. However, decades of non-systematic approached, variable interpretations, and application originating from guidelines developed in high-income settings, may have led to limited uptake in resource-constrained settings. Adequately supported (with training and capacity building) and implemented, contextualisation and adaptations of existing evidence-based recommendations may provide more cost-effective solutions to improving quality of care for people living with MSK conditions where the need is greatest. We therefore call on global health bodies, health ministry technical teams, professional societies, university departments, and guideline producing bodies such as NICE, OARSI, EULAR, ACR and G-IN to prioritise well-coordinated approaches to health systems strengthening in LMICs.

Though not specific to MSK, our findings corroborate that of a doctoral thesis including a comprehensive review of literature on PPI in clinical practice guidelines [35]. Beyond guideline development and dissemination, PPI and engagement in guideline implementation including improvements in health service delivery and care pathways is yet in its infancy, especially in low resource settings. Our findings emphasise the need to move away from tokenistic approaches towards evidence-based guidance partnership and ownership with patients, carers and the public.

Failed reporting culture could be another challenge or setback in shared learning and informing stakeholders’ communities about PPI in evidence-based guidance implementation activities. Articles reporting PPI implementation activities without specific reference to any MSK guideline or evidence-based recommendations were not included in this review. To this end we call for more targeted efforts to reporting in the literature, specific PPI activities in guideline implementation akin to the GRIPP2 recommendations.

## Limitations

In the review process many studies were excluded as they reported PPI in guidelines development process and research rather than implementation. We acknowledge however, that there is sometimes a blurred line between guidelines related research dissemination and actual implementation. Some reports could have therefore been missed. We therefore call the attention of academics, knowledge mobilisation professionals, funders and journal editors for more accurate reporting and labelling of implementation reports in the future.

As this article aims to present an overview of current evidence, restrictions to the design of primary studies as part of eligibility criteria for this review would have made it difficult to include any available evidence. Across included studies, there was a wide heterogeneity in the outcomes of PPI activities in MSK guideline implementation, precluding any form of quantitative synthesis. Consequently, we have taken a more cautious and descriptive approach to reporting of outcomes of PPI in guideline implementation.

Beyond the scope of this review, we acknowledge the need for a more detailed evaluation and review of evidence which may be better served by more robust methodological approach including data linkage, tracing and mapping. However, this could also be hampered by limited reporting of PPI activities. We call the attention of funding bodies to the need to invest more on implementation projects and research shaped by robust PPI, and PPI activities that are well reported.

### Future perspectives

For many healthcare conditions, available international evidence-based guidance is generated based on high-quality research with PPI, however, guideline impact varies widely and is highly contingent on successful transformation into practice. This review has been conducted with MSK guidelines as an exemplar field for PPI in evidence-based guidelines implementation including a focus on LMICs. Given, the acceptance of meaningful PPI in research, we propose that similar principles involving shared partnership and leadership may contribute to and inform more meaningful engagement and development of innovative, patient-centred implementation of evidence-based guidance for MSK and other conditions.

It will be particularly important for stakeholders (researchers, HCPs and PPI) to come together to establish and agree what guideline implementation should be in practice. This will form a basis for the reporting, evaluation of PPI in implementation. Communities of practice can then be formed to contextualise such standards in local settings.

Our PPI co-authors emphasise the need for a pathway to establishing and agreeing outcomes of consultations. They proposed “a preparing for your appointment type of meetings and leaflets” preferably lay-led, pre-clinical consultations to make patients aware of guidelines but also assist them and HCPs to work together, maximising consultation. This might also be helpful in low-resource settings were cultural contexts, power imbalances between patients, health literacy issues impact quality of care. It is our hope that this review will initiate and/or contribute to:

1. discussions regarding development of practical solutions for minimising the research-practice gap for MSK conditions globally,
2. highlight the need for maximising public partnership (beyond collaborations for health research) as a way to advance evidence-based guidance implementation
3. development of innovative models for advancing PPI in evidence-based guideline implementation and, consequently, enable swifter, broader uptake and more sustained use of best evidence in healthcare delivery.

## Conclusion

Whilst many clinical guidelines provide recommendations regarding best practice (i.e., what to do) for the care of MSK conditions, they often fail to address how to operationalise these recommendations into clinical practice. Evidence-based management of chronic MSK conditions moves beyond clinical settings where context is key. This review highlights knowledge, skills and practice gap for PPI in implementation of evidence-based guidelines for MSK conditions. The ‘Alliance conceptual framework for PPI in guideline implementation’ though subject to more formal development and refinement, is applicable to varying services/care pathways and can be relevant even in low resource settings. We call on relevant stakeholders to prioritise efforts to help to bridge the evidence-practice gap and to improve quality of care for musculoskeletal patients globally through novel partnerships together with PPI.

### Supplementary Information

**Additional file 1:** Plain English Summary.

## Data Availability

All data generated or analysed during this study are included in this published article [and its supplementary information files].

## Appendix 1: Full Medline search strategy (adapted for other databases)

1. Musculoskeletal Diseases/
2. (musculoskeletal or MSK).ab,kf,ti.
3. (chronic adj3 pain).ab,kf,ti.
4. ((multisite or "multi site") adj3 pain).ab,kf,ti.
5. pain syndrome$.ab,kf,ti.
6. (back adj3 pain*).ab,kf,ti.
7. (neck adj3 pain*).ab,kf,ti.
8. (shoulder adj3 pain*).ab,kf,ti.
9. (knee adj3 pain*).ab,kf,ti.
10. (joint adj3 pain*).ab,kf,ti.
11. exp Musculoskeletal Pain/
12. exp Back Pain/
13. Neck Pain/
14. knee pain.mp.
15. arthriti$.ab,kf,ti.
16. osteoarthr$.ab,kf,ti.
17. arthralgi$.ab,kf,ti.
18. Rheumatology/
19. rheumat$.ab,kf,ti.
20. (joint$ adj3 disease$).ab,kf,ti.
21. 1 or 2 or 3 or 4 or 5 or 6 or 7 or 8 or 9 or 10 or 11 or 12 or 13 or 14 or 15 or 16 or 17 or 18 or 19 or 20
22. Community Participation/
23. Patient Participation/
24. 22 or 23
25. (patient* adj1 (participat* or involv* or engag* or partnership or partners or collaborat* or consult*)).ab,ti.
26. (public adj1 (participat* or involv* or engag* or partnership or partners or collaborat* or consult*)).ab,ti.
27. (user* adj1 (participat* or involv* or engag* or partnership or partners or collaborat* consult*)).ab,ti.
28. (service user* adj1 (participat* or involv* or engag* or partnership or partners or collaborat* or consult*)).ab,ti.
29. (consumer* adj1 (participat* or involv* or engag* or partnership or partners or collaborat* or consult*)).ab,ti.
30. (lay adj1 (participat* or involv* or engag* or partnership or partners or collaborat* or consult*)).ab,ti.
31. (citizen* adj1 (participat* or involv* or engag* or partnership or partners or collaborat* or consult*)).ab,ti.
32. (carer* adj1 (participat* or involv* or engag* or partnership or partners or collaborat* or consult*)).ab,ti.
33. (caregiver* adj1 (participat* or involv* or engag* or partnership or partners or collaborat* or consult*)).ab,ti.
34. (customer* adj1 (participat* or involv* or engag* or partnership or partners or collaborat* or consult*)).ab,ti.
35. (client* adj1 (participat* or involv* or engag* or partnership or partners or collaborat* or consult*)).ab,ti.
36. (community* adj1 (participat* or involv* or engag* or partnership or partners or collaborat* or consult*)).ab,ti.
37. (stakeholder* adj1 (participat* or involv* or engag* or partnership or partners or collaborat* or consult*)).ab,ti.
38. ((patient* and public) adj1 (involv* or participat* or engag* or partnership or partners or collaborat* or consult*)).ab,ti.
39. (user led or user-led or lay control or user control).ab,ti.
40. ((representative* or patient representative* or patient advocate* or expert by experience or famil* or relative* or survivor*) adj1 (participat* or involv* or engag* or partnership or partners or collaborat* or consult*)).ab,ti.
41. ((patient* or consumer* or citizen* or advisory) adj1 board*).ab,ti.
42. ((patient* or consumer* or citizen* or advisory) adj1 group*).ab,ti.
43. ((patient* or consumer* or citizen* or advisory) adj1 panel*).ab,ti.
44. (citizen* adj1 (jury or juries)).ab,ti.
45. 24 or 25 or 26 or 27 or 28 or 29 or 30 or 31 or 32 or 33 or 34 or 35 or 36 or 37 or 38 or 39 or 40 or 41 or 42 or 43 or 44
46. Practice Guideline/
47. exp Health Planning Guidelines/
48. guideline$1.kf,ti.
49. guidance.kf,ti.
50. standards.kf,ti.
51. ((practice or treatment$ or clinical) adj standard).kf,ti.
52. recommendation$1.kf,ti.
53. ((practice or treatment$ or clinical) adj3 consensus).kf,ti.
54. (practice adj (guideline$1 or guidance or standard$1 or recommendation$1)).ab.
55. (clinical adj (guideline$1 or guidance or standard$1 or recommendation$1)).ab.
56. (treatment$ adj3 (guideline$1 or guidance or standard$1 or recommendation$1)).ab.
57. (CPG or CPGs).kw,ti.
58. Critical Pathways/
59. position statement$1.ab,kw,ti.
60. position statement$1.ab,kw,ti.
61. (practice adj3 parameter$1).ab,kw,ti.
62. (((critical or clinical or practice) adj3 (path$1 or pathway$1 or protocol$1)) and (guideline$1 or guidance or standard$1 or recommendation$1)).ab.
63. ((care adj3 (path$1 or pathway$1 or map$1 or plan or plans)) and (guideline$1 or guidance or standard$1 or recommendation$1)).ab.
64. ((care adj3 standard$1) and (guideline$1 or guidance or recommendation$1)).ab.
65. (("National Institute for Health and Care Excellence" or NICE) and (guideline$1 or guidance or recommendation$1)).ab,ti.
66. ((EULAR or "European League against Rheumatism") and (guideline$1 or guidance or recommendation$1)).ab,ti.
67. ((OARSI or "Osteoarthritis Research Society International") and (guideline$1 or guidance or recommendation$1)).ab,ti.
68. ((RCGP or "Royal College of General Practitioners") and (guideline$1 or guidance or recommendation$1)).ab,ti.
69. ((CSP or "Chartered Society of Physiotherapy") and (guideline$1 or guidance or recommendation$1)).ab,ti.
70. consensus development conference.pt.
71. 46 or 47 or 48 or 49 or 50 or 51 or 52 or 53 or 54 or 55 or 56 or 57 or 58 or 59 or 60 or 61 or 62 or 63 or 64 or 65 or 66 or 67 or 68 or 69 or 70
72. 21 and 45 and 71

## Abbreviations

MSK: Musculoskeletal
PPI: Patient and public involvement
LMICs: Low- and middle-income countries
WHO: World Health Organization
G-IN: Guidelines International
NICE: National Institute for Health and Care Excellence
OARSI: Osteoarthritis Research Society International
EULAR: European League Against Rheumatism (EULAR)
ACR: American College of Rheumatology
OA: Osteoarthritis
HCPs: Healthcare professionals
NIHR: National Institute for Health Research
GRAPPA: Group for research and assessment of psoriasis and psoriatic arthritis
