# Supplementary material for: Patient and public involvement in implementation of evidence-based guidance for musculoskeletal conditions: a scoping review of current advances and gaps
Source: BMC Rheumatol. 2022 Oct 22;6:84. doi: 10.1186/s41927-022-00310-x (PMC9588238; doi:10.1186/s41927-022-00310-x)
Supplement: Supplementary file 1 — Additional file 1: Plain English Summary. [file 41927_2022_310_MOESM1_ESM.docx]

| **What do we know about** **Patient and Public Involvement in Implementation of Evidence-Based Guidance for Musculoskeletal Conditions?** |
| --- |
| **Babatunde Opeyemi O*,**  **Linda Parton*, Dawson Shoba∞, Brammar June~, Dziedzic Krysia*, Adebajo Adewale O#** |

| Lay summary for: Patient and Public Involvement in Implementation of Evidence-Based Guidance for Musculoskeletal Conditions: A Rapid Review of Current Advances and Gaps.  March 2022 |
| --- |

**Key messages**

- Patient and public involvement (PPI) in research is more advanced than in implementation, (i.e., putting guidance into practice).
- There are evidence-based guidance recommendations to support care for patients with musculoskeletal (MSK) pain conditions. However, there are still differences in care being available and quality of care worldwide.
- As a result, many good research and evidence-based guidance are going to waste, without real benefits for patients with MSK, especially for people in developing countries.
- Currently available literature suggests PPI in putting guidance into practice consists mostly of low-level involvement activities.
- When good guidelines have been developed, sharing the findings is the first step but acceptance. However, continued use of the guidance in practice involves a lot more. Embracing patient views and ideas should help with this.
- Patient voice and input is important in encouraging use of evidence-based guidance among health professionals as well as patients and public.
- More should be done to encourage sharing of new knowledge and ideas about using MSK guidance, among local and international communities of PPI. This may also help to improve care including for those living with MSK patients in LMICs.

**Background: Why PPI in Implementation of Evidence-Based Guidance for Musculoskeletal Conditions?**

Public and patient involvement (PPI) can be defined as 'research being carried out 'with' or 'by' members of the public rather than 'to or for' them. Over time, PPI in research has promoted better quality research and better outcomes for patients. This is especially true for musculoskeletal pain conditions. Musculoskeletal conditions (MSKs) are injuries of the muscles, nerves, tendons, ligaments, joints, cartilage, or spinal discs. MSKs are not typically the result of any instantaneous or acute event (such as a slip, trip, or fall) but reflect a more gradual or chronic development.

Public and patients have been involved in many aspects of MSCs research from design to sharing findings. Increased PPI has also made MSCs guideline development more rigorous. However, there are still differences in care being available and quality of care for people with musculoskeletal conditions.

When good research has been done, and guidelines have been developed, sharing the findings is the first step but acceptance and continued use of the guidance in practice involves a lot more. This is called implementation. Patient voice is important in implementation, as health professionals may not know what it is like to live every day with a condition. All those involved in implementing findings should be working together with patients and the public to ensure information is accessible to all and is useable and used. Hopefully, this will lead to improvements in health service delivery.

**What did we want to find out?**

- We wanted to find out more about PPI in putting MSK guidelines into practice.
- We wanted to explore if PPI activity helps to improve health care delivery for people with MSK pain conditions.
- We also wanted to look for information about PPI activities in Lower- and Middle-Income Countries (LMICs).
- We wanted to see if any PPI, we found, helped with putting any guidelines into practice.
- We wanted to find out areas for future research on this topic.

**What did we do?**

We searched widely to find literature about PPI in putting guidelines into practice. We screened the reports, so we only looked at relevant ones. We extracted information about:

- Country where activities took place
- MSK condition
- Recruiting public and patients
- Details of PPI activities
- Outcomes

We looked at the different types of PPI activities reported. We mapped these to what we know about putting guidelines into practice. What we found has helped us to identify implications for putting guidelines into practice. We were also able to identify gaps in current literature and suggest areas for future research.

Throughout the research we worked closely with two public contributors who have experience of working with researchers to put guidance into practice.

**What did we find?**

Our first search gave us 1586 documents. We looked at 58 reports in full. We excluded many of these for various reasons including:

- PPI activities only contributed to guideline development or research
- PPI activities were not described in detail
- Some were not related to a specific MSK condition
- The report was not concerned with PPI in putting guidelines into practice

We ended up with a sample of 10 articles. These articles described public and patients, researchers and healthcare professionals working together to put guidance into practice. The articles were published between 2009 and 2020.

Most of the articles came from Europe with one coming from Asia (Turkey) and one from Africa (South Africa). The MSK conditions included arthritis, chronic musculoskeletal pain, and psoriasis.

We divided the PPI activities described into three levels:

- Involvement activities (2 reports)
- Consultation (4 reports)
- Shared partnership and leadership (4 reports)

We only had one report of PPI in putting guidance into practice in a LMIC. The PPI was low level.

We suggest key areas of focus for future practice and research:

- How can we improve PPI in putting guidance into practice?
- How can we share knowledge and experience between countries, including LMICs?
- How can we ensure continuity in PPI from research to developing guidelines and putting guidance into practice in communities?

The evidence is up to date to July 2021.

**What are the limitations of the evidence?**

We may have missed evidence of PPI in putting guidelines into practice for a number of reasons:

- Use of different words for ‘implementation’ and ‘PPI’ across the world
- Public health intervention reports related to MSK are not in the public domain
- Our research focussed on MSK conditions. There may be good examples of putting guidance into practice for other conditions.

Our findings need to be considered carefully.

**Remarks**

All the efforts and funds being used for research and developing guidelines will go to waste if these are not put into practice. We want to make sure guidance is used in real life to improve care for patients. Based on what we found in the literature, and our own experiences of working in this field, we created an illustration (the “Alliance” framework, Fig.1) of how PPI can help to support putting guidelines into real world practice. We know that information for improving care will continue to grow, and as we know more, new guidance will need to be added for improving practice. Therefore, we suggested continuous cycles of “thinking and doing” together with PPI, health professionals and those who are responsible for provision of care or organising health services.

We advocate for:

- PPI as key partners: the process of putting guidelines into practice will be a journey better together with patient voice.
- Settings is important – so organisation of care needs to embrace real world views, and funds available. PPI will be able to help with this.
- Sharing of new knowledge and ideas, among local and international communities of PPI will help to improve care including MSK patients in LMICs.

**What next?**

- There should be more, and better reporting of PPI activities for putting guidance into practice
- Our illustration (the “Alliance” framework) of how PPI can help to support putting guidelines into real world practice needs to be developed further.
- We should create communities of PPI who will share knowledge and ideas, for putting MSK guidance in practice. This should include people from LMICs.


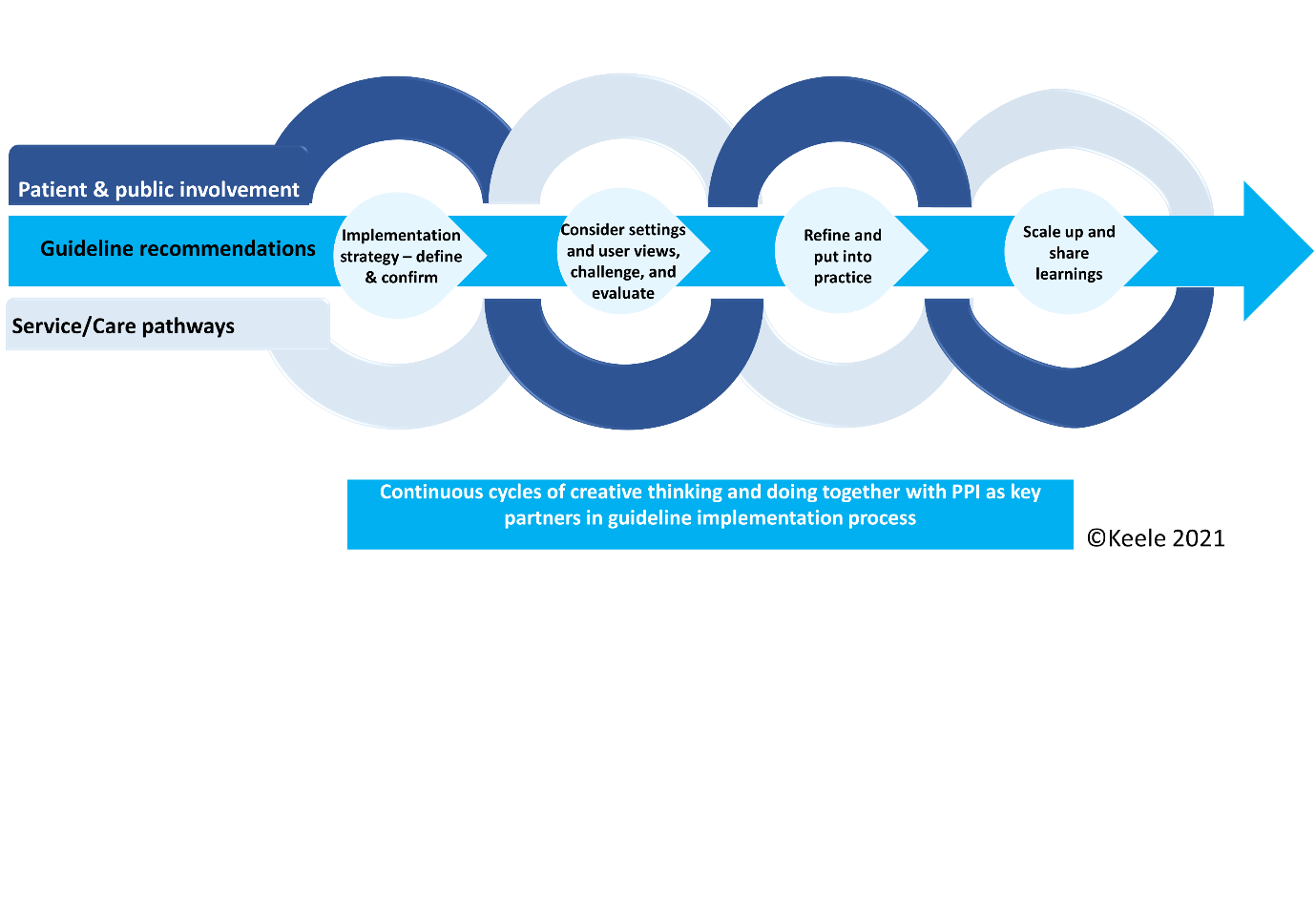


**Figure 1: The Alliance Framework: For PPI in evidence-based guidance implementation**
